# Case Report: Cognitive Conversion in a Non-brazilian VAPB Mutation Carrier (ALS8)

**DOI:** 10.3389/fneur.2021.668772

**Published:** 2021-06-02

**Authors:** Anna G. M. Temp, Martin Dyrba, Elisabeth Kasper, Stefan Teipel, Johannes Prudlo

**Affiliations:** ^1^German Center for Neurodegenerative Diseases (DZNE), Rostock, Germany; ^2^Department of Neurology, Rostock University Medical Center, Rostock, Germany; ^3^Department of Psychosomatic Medicine, Rostock University Medical Center, Rostock, Germany

**Keywords:** ALS8, case report, cognition, MRI, volumetric change

## Abstract

Amyotrophic lateral sclerosis 8 (ALS8) is a predominantly lower motor neuron syndrome originally described in a Portuguese–Brazilian family, which originated from a common founder. ALS8 is caused by a VAPB mutation and extremely rare in Central Europe. We present a 51-year-old German man with ALS8 who had the P56S VAPB mutation independently of the founder effect. In the final 4 years of his life (disease duration 10 years), the patient had five MRI scans and four in-depth neuropsychological assessments. This paper addresses the course of the patient's cognitive status and relates cognitive performance to structural brain changes in order to determine whether this ALS8 case showed a different pattern of cognitive decline compared with sporadic ALS. The executive functions, verbal fluency, and memory of the patient and 17 age-, sex-, and education-matched controls were assessed on four different occasions. His cognitive performance and decline were investigated for abnormality using cross-sectional and longitudinal matched case–control analysis. We obtained five T1-weighted MRI, which we analyzed using voxel-wise non-parametric analysis with *statistical non-parametric mapping* in Matlab. Moreover, we conducted a single-subject correlation between cognitive performance and brain atrophy. The cognitive profile of the index patient featured executive dysfunction. Notably, his working memory and shifting ability declined from a healthy baseline to an impaired performance, leading to a transition from cognitively non-impaired (ALSni) to cognitively impaired (ALSci). The correlations we observed between cerebellar atrophy and verbal fluency in addition to fusiform gyrus atrophy and shifting are novel findings. We found that the conversion from ALSni to ALSci was associated with widespread cerebral atrophy, which extended beyond the primary motor and premotor cortex and affected, among others, the cerebellum and left fusiform gyrus. The index patients' cognitive profile resembles that of other ALS phenotypes, but the extensive atrophy beyond extra-motor areas has not yet been described.

## Introduction

Amyotrophic lateral sclerosis 8 (ALS8) is an autosomal-dominantly inherited form of familial ALS, first described clinically as a late-onset “progressive muscular atrophy” in a Portuguese–Brazilian family in 1962 (1). ALS8 is caused by a VAPB mutation (2). The endoplasmic reticulum (ER)-resident gene product, the vesicle-associated membrane protein-associated protein B (VAPB), is a vesical fusion protein and involved in the ER unfolded protein response (3). Haplotype analyses support the assumption of a common founder during the beginning of the Portuguese colonization of Brazil in the mid-15th century (4). ALS8 is extremely rare in Central Europe: among 301 ALS patients screened for genetic mutations as part of one study, none had this mutation (5).

**Figure 1:**
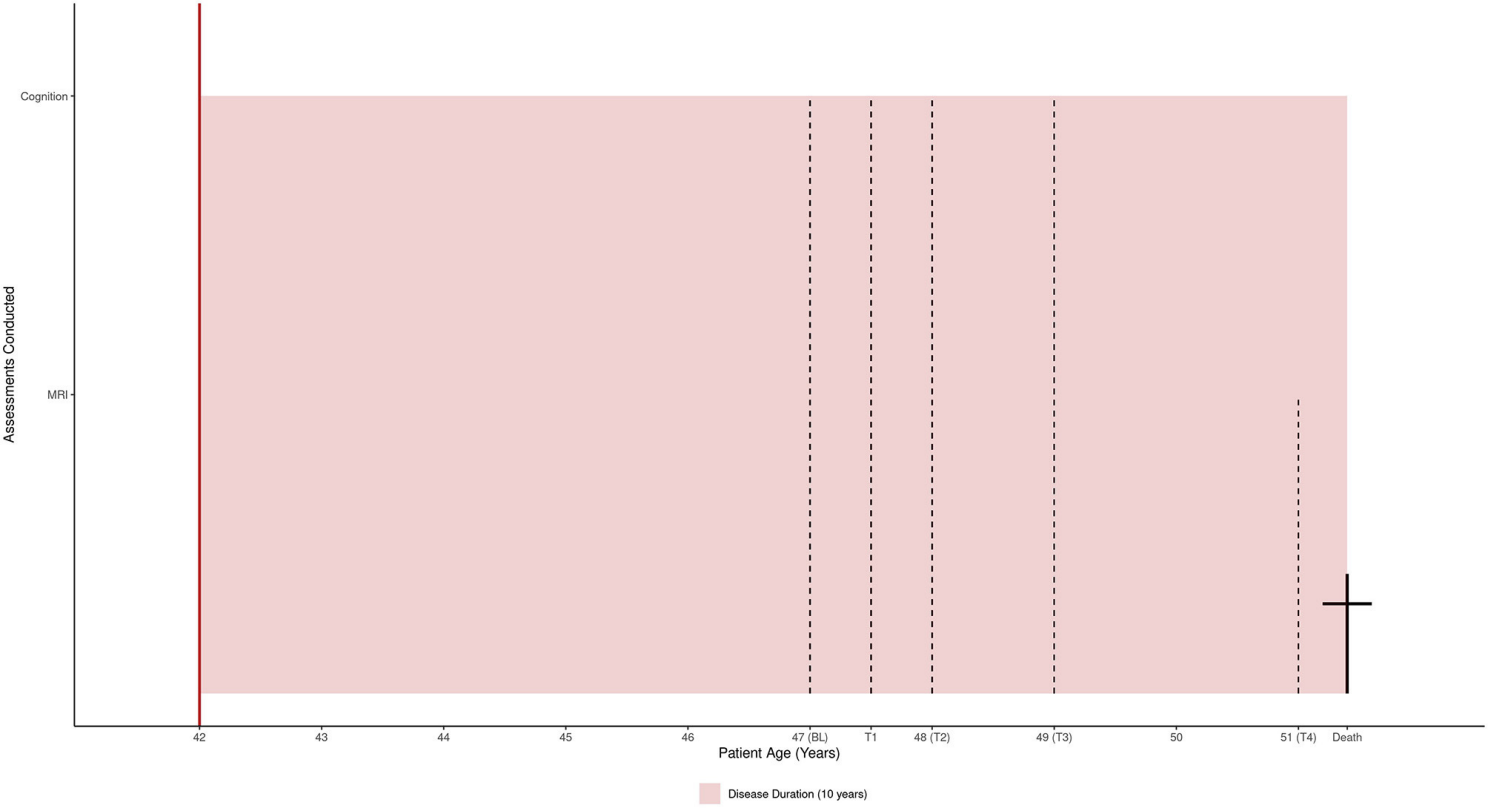
The patient's assessments and 10-year disease duration mapped to his life span (51 years).

ALS8 is a predominantly lower motor neuron syndrome. Comparable to sporadic ALS, approximately a third of ALS8 patients are cognitively impaired (6), although an association with frontotemporal dementia has not yet been described. De Alcantara et al. (6) examined the cognition of 22 Brazilian ALS8 patients (14 men) compared to healthy controls. They investigated episodic memory, executive functions, emotion recognition, behavior, mood, and global cognitive functioning using the MMSE. These ALS8 patients showed deficits in semantic fluency (but not letter fluency), digit span forward, similarity finding, and sentence completion. These deficits strongly suggest that the cognitive profile of ALS8 patients is predominantly characterized by executive dysfunction comparable to the cognitive profile of patients with sporadic ALS. In the current single case study, we present a 51-year-old German man with ALS8 who had the P56S mutation without the Portuguese–Brazilian haplotype, independently of the founder effect (7). In the final 4 years of his life (disease duration 10 years), the patient described here had longitudinal assessment with five MRI scans and four in-depth neuropsychological examinations (see Figure 1). We describe the longitudinal course of the patient's cognitive status and structural brain changes compared with age- and sex-matched healthy people. We wanted to determine whether the patient declined cognitively, whether cognitive changes were associated with gray matter atrophy, and where gray matter atrophy occurred. Comparing these findings with reported changes in sporadic ALS would allow us to assess similarities and differences between this VAPB carrier case and sporadic ALS in the profiles of cognitive decline and gray matter atrophy.

## Materials and Methods

### Case Presentation

The primary case was a German man with ALS8. His known family history included seven afflicted family members in three generations; their details can be found in Funke et al. (7). The patient was 41 years old when his symptoms began and died at the age of 51. He had completed 13 years of formal education. Based on his verbal-crystallized intelligence, estimated at baseline, his pre-morbid IQ was in the upper average range (114 points).

### Neurological and Motor Deficits

The first signs of ALS appeared in the form of a lower motor neuron (LMN) syndrome in the lower limbs. Initially, the 41-year-old patient described having experienced premature exhaustion while running a marathon, followed by weakness of dorsiflexion of the ankles (first right, then left) and recurrent falls. Four years later, weakness of the legs affected the patient in the following ways: walking without assistance was reduced to 50 m, walking with a cane was reduced to 400 m, and rising from a squatting to standing position had become hardly possible, necessitating a wheelchair at 45 years of age. Proximal weakness in the upper limbs started from the 4th year of the disease. In contrast to the lumbosacral region with only LMN signs, the cervical region showed evidence of both upper and LMN dysfunction, thus meeting the definition of *classical* ALS. Although bulbar signs were evident, bulbar symptoms (excluding mild dysphagia) were without significance throughout the course of the disease. There was no weight loss and no clinical signs of compromised respiratory muscle function. Initially, the patient had an ALS functional rating scale-revised (ALSFRS-R) score of 35/48 (8). Thirty-nine days prior to his death, his final imaging appointment took place (see Figure 1) at which his ALSFRS-R was 30/48. The patient was a slow progressor (ALSFRS-R δ = 0.11), with a loss of five points over 45 months. He met the El Escorial criterion for *probable*, laboratory-confirmed ALS (9). As he rejected an autopsy, the cause of death could not be precisely determined, but he was considered to have died a natural death. At no point did he require non-invasive ventilation, tracheotomy, or percutaneous endoscopic gastrostomy.

### Patient Perspective

The patient was very keen to participate in research as he viewed it as serving the public by contributing to an improved understanding of ALS8. He derived a sense of purpose from his participation. In his coping with the disease, he was determined to retain his quality of life and independence and proactively devised strategies to obtain these goals. In this, he maintained a realistic view of his own abilities and disabilities, and insight into his disease progression. He showed no signs of disease catastrophizing.

### Control Data

We obtained neuropsychological data from 17 suitable men from the control group of our cohort study (10, 11, also in Supplementary Table 1). These men contributed data at baseline and 6 months later, at T1. While it would have been informative to conduct additional comparisons to sporadic ALS patients, this was not feasible due to the heterogeneous nature of the disease: sporadic ALS may present with and without cognitive–behavioral changes (10–13), and sporadic ALS patients would need to be matched for spinal onset site and disease duration as well as age, sex, and education. None of the male patients in our cohort (14, 15) were suitable due to the patient's unusually long disease duration [10 years, (16)] and high pre-morbid IQ. Comparisons to non-VAPB forms of ALS will thus be accomplished by comparing our findings with reported findings in the literature, rather than by statistical analysis.

### Measures

Details on our neuropsychological battery have been published elsewhere (14, 15). We selected those assessments which were completed by our patient; see Tables 1, 2 and Supplementary Table 3.

**Table 1:** The patient's cognitive abilities at his baseline neuropsychological examination.

**Assessment**	**Healthy controls**			**The patient**	**Two-tailed Bayesian hypothesis test**	**Estimated percentage of the controls obtaining a lower score than the patient**		**Estimated effect size (z**_**HC**_**)**	
	***N***	**Mean**	**SD**		***p*-value**	**Point**	**95%CI**	**Point**	**95%CI**
MoCa	17	28	2	27	0.634	31.68	15.93 to 50.51	−0.50	−0.99 to 0.01
Digit span fw.	17	8	2	8	0.999	50.01	31.74 to 68.29	0.00	−0.48 to 0.48
Digit span bw.	17	6	2	5	0.634	31.69	15.93 to 50.51	−0.50	−0.99 to 0.01
Learning	17	50	7	50	0.999	50.01	31.74 to 68.29	0.00	−0.48 to 0.48
Recall 1	17	11	3	10	0.750	37.51	20.70 to 56.38	−0.33	−0.82 to 0.16
Recall 2	17	10	2	9	0.633	31.69	15.93 to 50.52	−0.50	−1.00 to 0.01
Recognition	17	9	4	10	0.811	59.45	40.64 to 76.72	0.25	−0.24 to 0.73
Phon. VF (Σ)	17	13	4	15	0.633	68.32	49.48 to 84.08	0.50	−0.01 to 1.00
sem. VF (Σ)^*^	**17**	**24**	**5**	**35**	**0.048**	**97.58**	**90.26 to 99.90**	2.20	**1.30 to 3.08**
TMT B/A	17	2.16	0.65	1.67	0.474	23.73	9.93 to 41.96	−0.75	−1.29 to −0.20

* p < 0.05, **p < 0.01, ***p < 0.001.

**Table 2:** At his final follow up, the patient's neuropsychological examination showed executive impairments.

**Assessment**	**Healthy HC**			**The patient**	**Two-tailed Bayesian hypothesis test**	**Estimated percentage of the HC obtaining a lower score than the patient**		**Estimated effect size (z**_**HC**_**)**	
	***N***	**Mean**	**SD**		**Probability**	**Point**	**95%CI**	**Point**	**95%CI**
MoCa	17	29	2	29	0.999	50.00	31.74 to 68.29	0.00	−0.48 to 0.48
**Digit Span fw**.^**†**^	**17**	**8**	**1**	**6**	**0.070**	**3.49**	**0.24 to 12.41**	**−2.00**	**−2.82 to** **−1.16**
Digit Span bw.^**^^,^^†^	**17**	**7**	**1**	**2**	** <0.001**	**0.01**	**0.00 to 0.07**	**−5.00**	**−6.77 to** **−3.21**
Learning	17	51	7	52	0.891	55.44	36.80 to 73.20	0.14	−0.34 to 0.62
Recall 1	17	11	3	12	0.750	62.50	43.63 to 79.32	0.33	−0.16 to 0.82
Recall 2	17	11	3	11	0.999	50.01	31.74 to 68.29	0.00	−0.48 to 0.48
Recognition	17	12	3	12	0.999	50.01	31.74 to 68.29	0.00	−0.48 to 0.48
Phon. VF (Σ)	17	15	3	21	0.070	96.51	87.57 to 99.76	2.00	1.15 to 2.82
Phon. VF (Index)	17	3.4	0.84	2.19	0.180	9.04	1.72 to 22.88	−1.44	−2.11 to −0.74
sem. VF (Σ)	17	21	5	27	0.261	86.97	71.21 to 96.54	1.20	0.56 to 1.82
sem. VF (Index)	17	2.21	0.62	2.19	0.976	48.78	30.61 to 67.15	−0.03	−0.51 to 0.44
TMT B/A^*^^,^^†^	**17**	**2.31**	**0.61**	**3.79**	**0.031**	**98.43**	**92.74 to 99.96**	**−2.43**	**1.46 to 3.37**
ToL correct	17	4	1	4	0.999	50.01	31.74 to 68.29	0.00	−0.48 to 0.48
ToL errors	17	0	1	0	0.999	50.01	31.74 to 68.29	0.00	−0.48 to 0.48
ToL moves	17	37	4	37	0.999	50.01	31.74 to 68.29	0.00	−0.48 to 0.48

* *p < 0.05*,

** *p < 0.01*,

*** *p < 0.001*,

† *impaired function*.

### Procedure

Details of the data collection procedure are available from Kasper et al. (14, 15). Figure 1 maps the patient's life span and assessments to his disease duration (10 years).

### Ethical Considerations

The patient consented to contribute his imaging data in addition to his neuropsychological assessments to our research studies (14, 15). The local medical ethics committee approved this study (A2010-32 and A2011-56).

### MRI Acquisition

MRI scanning was performed in all 18 cases and at every time point with a single 3T Siemens Magnetom VERIO scanner (Erlangen, Germany) using a 32-channel head coil. High-resolution T_1_-weighted anatomical images were acquired using the magnetization-prepared rapid gradient echo (MPRAGE) sequence with the following parameters: 256 × 256 image matrix with 192 sagittal slices, FOV 250 × 250 × 192 mm, voxel size 1 × 1 × 1 mm^3^, echo time 4.82 ms, repetition time 2,500 ms, and flip angle 7°.

### MRI Data Preprocessing

The anatomical T_1_-weighted images from all time points were co-registered to each other, segmented into gray matter, white matter, and cerebrospinal fluid partitions using the CAT12 toolbox longitudinal pipeline in Matlab 2019ab. Then, the *Diffeomorphic Anatomical Registration Through Exponentiated Lie (DARTEL)* algebra algorithm (17) was used in combination with the default CAT12 brain template to normalize the individual mean T_1_-weighted image to the *Montreal Neurological Institute (MNI)* reference coordinate system. The estimated individual deformation field was applied to the gray matter segments of all time points to bring them into the MNI space as well, followed by modulation to preserve the total amount of gray matter and smoothing with an 8-mm Gaussian kernel.

### Statistical Considerations

We addressed the first research question—whether the patient experienced cognitive decline—using the programs *SingleBayes_ES.exe* and *DiffBayes.exe* (18). *SingleBayes_ES.exe* produces a Bayesian probability value, a point estimate of the percentage of HC who performed worse than our patient (“point estimate of abnormality” including a 95% credibility interval) and an effect size, z_HC_ (including a 95% credible interval). *Z*_*HC*_ estimates the average difference of our patient from the HC in standard deviation units. It is independent from the size of the control sample and the assessments used, thus expressing the patient's strengths and deficits on a shared metric. *Credibility intervals*—opposed to confidence intervals—provide us with a 95% probability that the true effect size will fall between the given bounds (18, 19). *DiffBayes.exe* was applied to quantify the progression in only those cognitive domains where the patient showed an impairment. It produces z_dHC_, the effect size for the difference between control decline and case decline, alongside a point estimate of abnormality. The patient's cognitive performance was considered impaired if measures from at least three tests fell below z_HC_ ≤ −1.5 (20, 21). This boundary was applied regardless of statistically significant differences from the HC.

The second and third questions—if any cognitive changes correlate with longitudinal neuroanatomical changes, and which neuroanatomical changes occurred regardless of cognition—were investigated using *SnPM13* (22) in Matlab 2019a. We used a single-subject correlation between any declining cognitive functions and neuroanatomy with 120 permutations, thresholding significance at *p* ≤ 0.05, and cluster size k ≥ 50 voxels.

## Results

Descriptive data are included alongside the inferential data in Tables 1, 2. Significant impairments of *p* ≤ 0.05 or z_HC_ ≤ −1.5 are printed in **bold**.

### Cognitive Decline

At baseline, the index patient's cognitive abilities were intact (see Table 1); his semantic verbal fluency was even better than those of the HC (*p* = 0.048, z_HC_ = 2.20 [1.30, 3.08]). At this time, his reading speed had not been recorded, so it was not possible to calculate verbal fluency indices for the patient's BL and the sum of all words produced (Σ) was instead analyzed. He met the criteria for ALS without cognitive or behavioral impairment [ALSni (13)].

**Figure 2:**
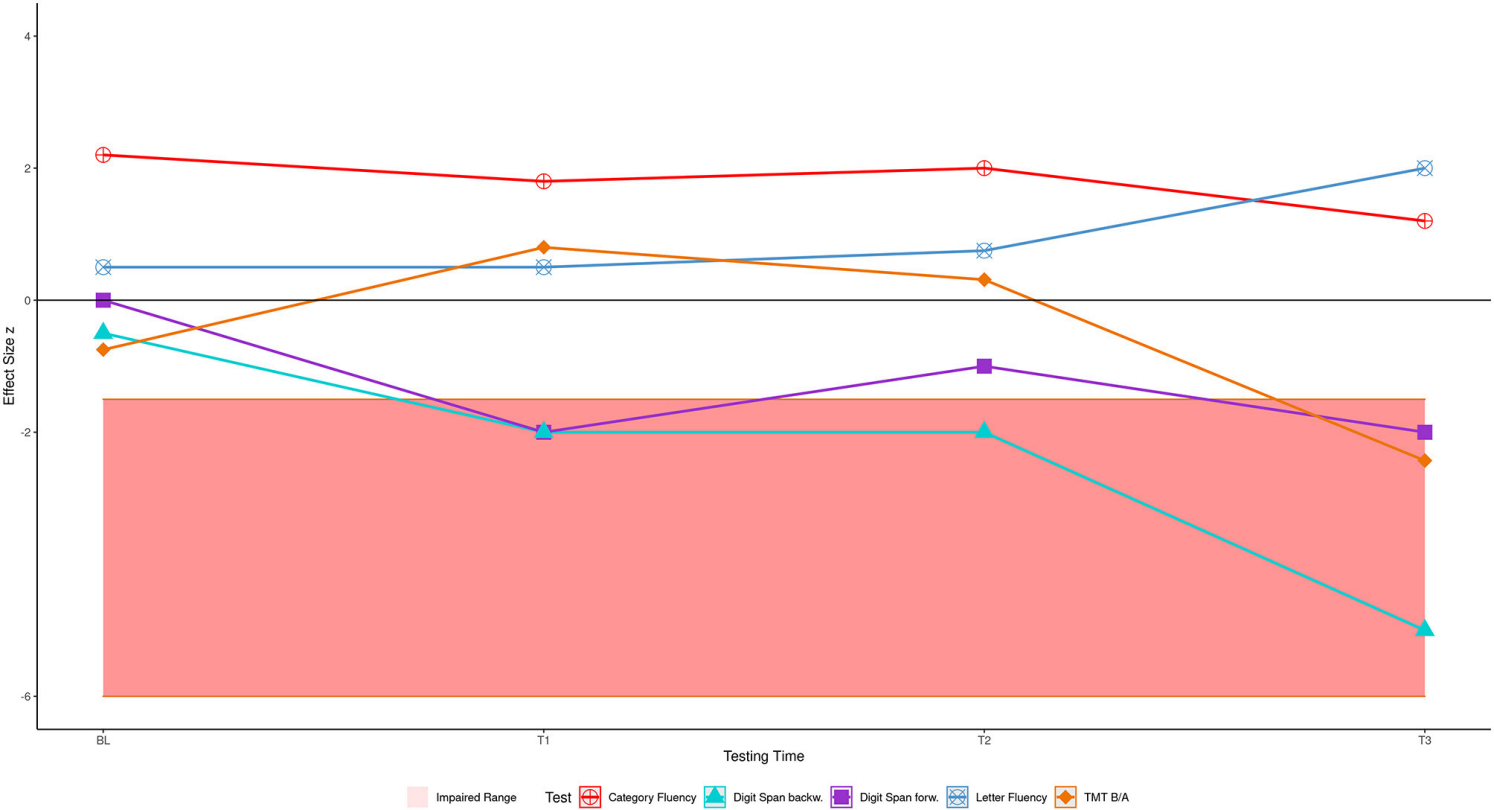
The patient's cognitive performance over time, shaded area shows impaired range.

Four years later (T3, in Table 2), several of his abilities had declined so that he now showed an impairment compared to the HC. In this final year of his disease, he met the Strong criteria for ALS with cognitive impairment because two non-overlapping executive functions were impaired: verbal working memory and cognitive flexibility. His semantic verbal fluency was no longer different from the HC; it had declined from an above-average performance (z_HC_ = 2.20 at BL) to an average performance of z_HC_ = 1.20 at T3. The patient's digit span performance declined more than was to be expected given the HC performance (*p* < 0.001, z_dHC_ = 6.22 [13.89, 8.23], point estimate = 0.00 [0.00, 0.01]). The patient's TMT B/A performance also declined (*p* = 0.020, z_dHC_ = −2.27 [−3.28, −1.36], point estimate = 1.99 [0.05, 8.75]). The decline of his semantic verbal fluency was non-significant (*p* = 0.291, z_dHC_ = 1.6 [0.23, 2.17], point estimate = 14.57 [1.50, 40.86]). The cognitive fluctuations are depicted in Figure 2. The answer to our first research question is therefore that the patient's shifting ability, short-term, and working memory were initially intact but declined until he exhibited a marked deficit.

### Correlations Between Cognition and Brain Volume

The neuropsychological results suggested a significant decline in both digit spans and TMT ratio, but non-significant change in VF. These four subtests were used as covariates in single-subject correlations between cognition and neuroanatomy. Supplementary Table 3 reports clusters larger than 200 voxels which significantly correlated with TMT ratio or semantic VF; no other correlations emerged.

**Figure 3:**
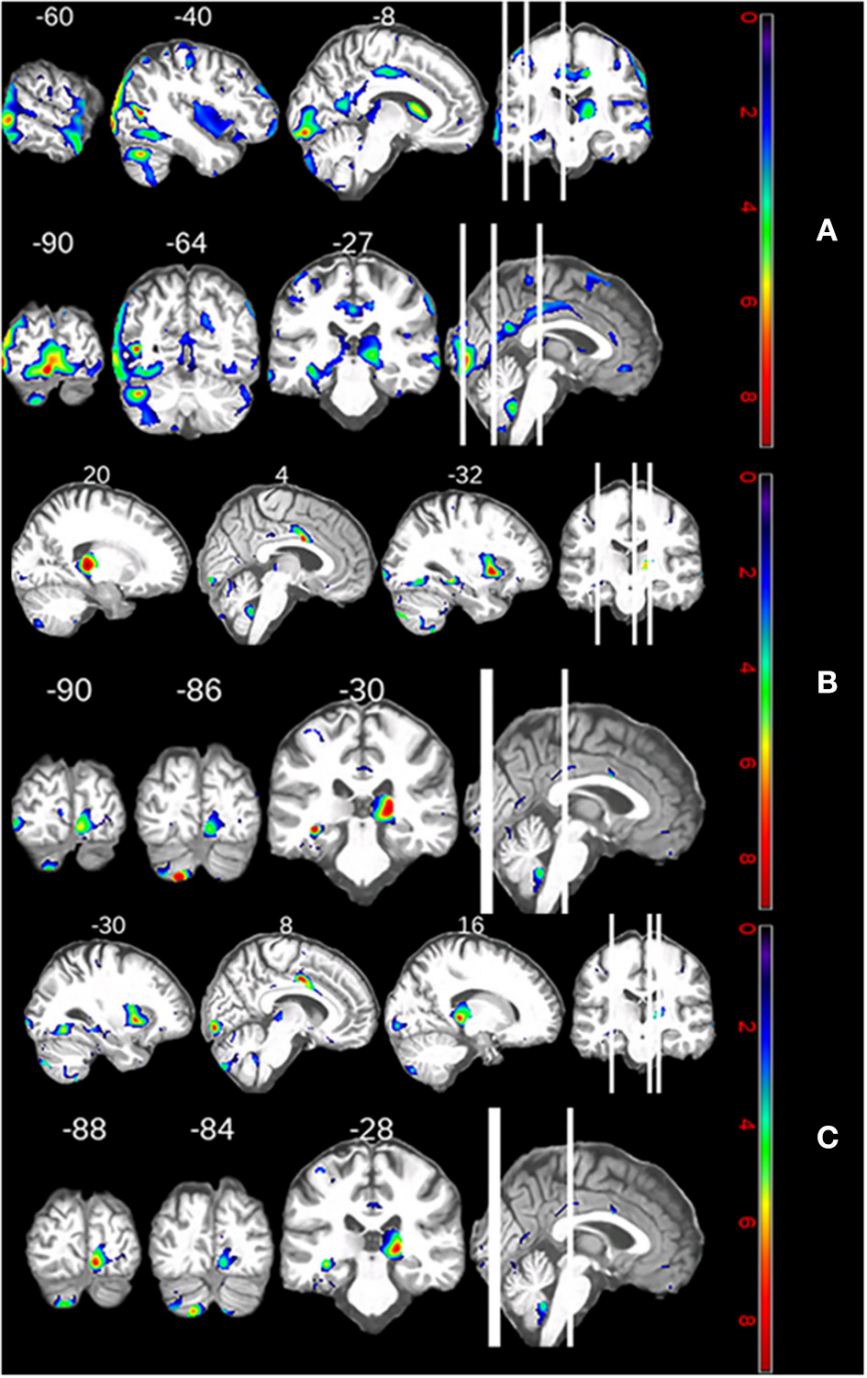
**(A)** Neuroanatomical correlates of the patient's declining shifting performance. **(B)** Neuroanatomical correlates of the patients' declining semantic fluency performance. **(C)** Regional increases in atrophy over 5 years.

Supplementary Table 3 shows that similar structures correlated with TMT ratio and semantic VF: the right cingulate and lingual gyri, as well as the left putamen, hippocampus, cuneus, and cerebellar crus II. The only structures which differentially affected the TMT ratio and semantic VF were the right hippocampus and left fusiform gyrus, and the right thalamus, respectively. Figures 3A,B illustrate these areas' atrophy. The depicted slice numbers correspond to the MNI coordinates in Supplementary Table 4 (the patient's left hemisphere continuously corresponds to the images' left side, pseudo-t range from 0 to 8).

We can answer our third research question: declining shifting ability and semantic processing correlated with atrophy in the cingulate and lingual gyri, as well as the left putamen, hippocampus, cuneus, and cerebellar crus II.

### Neuroanatomical Changes Over Time

The top three clusters of atrophy over time with regard to pseudo *t*-values were all located in the calcarine and pericalcarine cortices (see Supplementary Table 4). There was also atrophy in the left medial temporal gyrus, cerebellar crus II, caudate, and fusiform gyrus. Right-hemispherically, there was atrophy in the gyrus triangularis and the cerebellar tonsil. Functionally, these suggest impairments in the visual domain, semantic and memory processing, motor coordination, goal-oriented behavior, and face recognition (see Figure 3C).

## Discussion

We analyzed the cognitive and neuroanatomical changes in a 51-year-old German man with the P56S VAPB mutation, who, following a disease duration of 10 years, died of unexplained causes. He had a predominantly lower motor neuron syndrome with subtle bulbar symptoms. His 45-month assessment began 6 years after disease onset and ended 1 month prior to his death. His cognition deteriorated 8 years after the onset of the disease from cognitively non-impaired (ALSni) to cognitively impaired (ALSci) in accordance with the latest Strong criteria (13). At this time (2 years prior to his death), his shifting ability, working memory, and short-term memory showed impairments. However, no letter fluency impairment was detected; this is congruent with previous findings in ALS8 (6). Having been estimated to have had a high pre-morbid cognitive reserve, his semantic verbal fluency, although showing a decline, remained above the expected average. Intact verbal fluency is present in up to 50% of ALS cases in general (10). Impaired verbal fluency is common among cognitively impaired sporadic ALS patients, and thus not specific to ALS8. Having established his cognitive profile by comparison to healthy controls, we can contrast it with the existent literature on non-VAPB ALS.

The notion of cognitive stability in ALS is a matter of debate: whereas some groups have presented findings showing that the cognitive status of ALS patients remains relatively stable (14, 23–25), others have demonstrated mild cognitive decline as a frequent occurrence in sporadic ALS over time and in late stages of the disease (26–29). Consistent with the latter findings, here, we observed a transition from ALSni to ALSci in a case of familial ALS8.

In our patient, correlations between cognitive decline and atrophy were observed with regard to shifting impairment and semantic fluency decline. Shifting impairment was associated with the cingulate gyrus, hippocampus, and lingual gyrus of the right hemisphere, in addition to the putamen, cuneus, cerebellum, hippocampus, and fusiform gyrus of the left hemisphere. Semantic fluency decline was associated with the thalamus, cingulate gyrus, and lingual gyrus of the right hemisphere, and the cerebellum, hippocampus, putamen, and inferior occipital gyrus of the left hemisphere. Comparable atrophy of the cingulate and fusiform gyri has been observed cross-sectionally in a sample of cognitively impaired ALS patients (30), although that study did not correlate atrophy and cognition directly. Although the fusiform gyrus contributes to visual word recognition and orthographic processing (31, 32)—both skills necessary to solving the trail making test (TMT)—our longitudinally correlated structural and functional changes are yet to be established in non-VAPB ALS.

We observed semantic fluency decline with cerebellar atrophy in a similar manner to how cerebellar damage has been associated with verbal fluency deficits cross-sectionally in a non-ALS context (33). Stroke-damaged bilateral anterior thalamic radiation and left inferior fronto-occipital fasciculus tracts have also been associated with impaired verbal fluency (34). This suggests that our patient's cognitive and morphological changes may be shared between ALS8 and non-VAPB ALS. The *cerebellar cognitive affective syndrome* (35) presents with deficient task shifting and working memory, similar to our findings. As dysexecutive functioning is more typically associated with atrophy of the pre-frontal cortex, it was unusual that we found no frontotemporal reduction associated with our patient's executive dysfunction, specifically impairments of working memory and shifting (36–38). Frontotemporal atrophy occurs with and without cognitive impairments but is more severe when impairments are present (39–42). In contrast, we did not detect any frontal volume changes over 5 years and only very little temporal volume reduction associated with a decline in cognitive functioning. Curiously, working memory decline did not correlate with atrophy in any brain region. Given that the digit span task we applied is strongly related to general intelligence *g* (43), and that the patient had high levels of verbal-crystallized intelligence recently shown to protect working memory from regional atrophy (44), it is conceivable that his high cognitive reserve obscured the association between working memory performance and atrophy.

We observed increasing atrophy in both cerebellar hemispheres, the right pars triangularis, the left calcarine and pericalcarine cortices, the middle temporal gyrus, the caudate nucleus, and the left fusiform gyrus over a period of 5 years (see Figure 3C). This longitudinal change is congruent with cross-sectional studies which document atrophy in the calcarine cortex, superior parietal lobe, and putamen of cognitively unimpaired non-VAPB ALS (41, 45). Atrophy of the caudate nucleus has been described in two cross-sectional ALS samples: rapidly progressing sporadic ALS patients in addition to ALS patients who had not been cognitively or genetically characterized (46, 47).

A recent comprehensive longitudinal study on non-VAPB ALS patients documented progressive volume reduction in the bilateral amygdala, hippocampi, caudate, left accumbens nucleus, and right putamen of cognitively impaired ALS patients. Hippocampal and left caudate volumes also decreased over a period of 5 months (48). Our patient's atrophy patterns are therefore not unique to ALS8. However, unusual atrophy patterns underlay his cognitive deficit profile as the associations were less fronto-temporal and more temporo-cerebellar.

Rigorous statistical modeling techniques suitable to single cases ensured the internal validity of our conclusions; however, the facts that this was a single-case study and social cognition was not assessed are core limitations of our study. Our findings require further replication in a larger ALS8 cohort.

In conclusion, this longitudinal case study reveals that this non-Brazilian VAPB-mutation carrier showed more similarities to non-VAPB ALS than differences. His cognitive profile featured executive dysfunctioning. Notably, his working memory and shifting ability declined from a healthy baseline to an impaired performance, leading to a conversion from ALSni to ALSci, with his patterns of atrophy resembling previously documented atrophy patterns in non-VAPB ALS. The correlations we observed between cerebellar atrophy and verbal fluency and fusiform gyrus atrophy and shifting are novel findings requiring replication. We showed that the transition from ALSni to ALSci was associated with widespread posterior cerebral atrophy, which was unexpectedly observed outside of the primary motor cortex and/or the frontal cortices in this case, in contrast to common (non-VAPB) ALS.

## Data Availability Statement

The datasets presented in this article are not readily available because this is a case report; to protect anonymity not just of the patient but also his relatives the data must be kept confidential. Requests to access the datasets should be directed to Johannes Prudlo, johannes.prudlo@med.uni-rostock.de.

## Ethics Statement

The studies involving human participants were reviewed and approved by Ethikkommission an der Medizinischen Fakultät der Universität Rostock St.-Georg-Str. 108 18055 Rostock. The patients/participants provided their written informed consent to participate in this study. Written informed consent was obtained from the individual(s) for the publication of any potentially identifiable images or data included in this article.

## Author Contributions

AGMT conceptualized the neuropsychological single-case data analysis strategy, conducted the analysis and interpreted the neuropsychological results, conceptualized the neuroanatomical data analysis strategy, and drafted the manuscript. MD conceptualized the neuroanatomical data analysis strategy, conducted the analysis and interpreted the results, and revised the manuscript. EK conceptualized the neuropsychological assessment battery, collected the neuropsychological data, and revised the manuscript. ST consulted on the study design and analytical approach for neuroanatomy and revised the manuscript. JP acquired the funding, conceptualized the overall study approach, and revised the manuscript. All authors contributed to the article and approved the submitted version.

## Conflict of Interest

The authors declare that the research was conducted in the absence of any commercial or financial relationships that could be construed as a potential conflict of interest.
