# Supplementary material for: Case Report: Cognitive Conversion in a Non-brazilian VAPB Mutation Carrier (ALS8)
Source: Front Neurol. 2021 Jun 2;12:668772. doi: 10.3389/fneur.2021.668772 (PMC8208309; doi:10.3389/fneur.2021.668772)
Supplement: Supplementary file 1 [file Table_1.DOCX]

Supplementary Material for “Case report: cognitive conversion in a non-Brazilian VAPB mutation carrier (ALS8”

# Demographic and Study Background Data

The index patient, and his controls’ demographic background is provided in Table 1.

| Table 1. The Demographic Background of All Participants. | | | |
| --- | --- | --- | --- |
| Demographic Variable | Index Patient | Healthy Controls (HC) |  |
| N | 1 | 17 |  |
| Age at BL | 48 | 47 (3) |  |
| Years’ Education | 13 | 13 (2) |  |
| Premorbid IQ | 114 | 99-110 |  |
| Handedness | Right | 15 right, 2 unknown |  |

Additional information about the study’s cognitive tests, their respective domains and measurements can be found in Table 2.

| Table 2. Overview of the Cognitive Assessments and Domains. | | |
| --- | --- | --- |
| **Test Name** | **Measure** | **Cognitive Domain** |
| Montreal Cognitive Assessment (MoCA) | Total | Global |
| Trail Making Test | TMT A | Processing Speed |
|  | TMT B | Shifting |
|  | TMT B/A* | Shifting |
| Wechsler Memory Scale Revised | Digit span forw. | Short-Term Memory |
|  | Digit span backw. | Working Memory |
| Verbal Learning and Memory Test (VLMT) | VLMT 1-5 | Learning |
|  | VLMT 6 | Immediate Free Recall 1 |
|  | VLMT 7 | Delayed Free Recall 2 |
|  | VLMT WF | Recognition |
| Regensburg Verbal Fluency Test | Phonematic Fluency | Verbal Fluency |
|  | Phonematic Fluency Index* |  |
|  | Semantic Fluency |  |
|  | Semantic Fluency Index* |  |
| Tower of London | Tower of London Correct | Problem Solving |
|  | Tower of London Errors |  |
|  | Tower of London Moves |  |
| *Reflect tempo-adjusted indices to account for motor impairment (1). | | |

# Supplementary Results Tables

Table 3 shows the pseudo-t values, MNI coordinates and structures associated with declining TMT ratio or semantic verbal fluency.

| Supplemental Table 3. Overview of the Correlations between Regional Atrophy and TMT Ratio or Semantic VF. | | | | | | | | |
| --- | --- | --- | --- | --- | --- | --- | --- | --- |
| ***TMT Ratio*** | | | | | | | | |
| ***k*** | **Pseudo-t** |  |  |  | **MNI (x,y,z)** | | **Hemisphere** | **Structure** |
| 517 | 8.35 |  |  |  | 9, -3, 40 | R | | Cingulate gyrus |
| 1248 | 7.57 |  |  |  | 20, -32, 3 | R | | Hippocampus |
| 1584 | 7.15 |  |  |  | -33, 8, -2 | L | | Putamen |
| 730 | 6.59 |  |  |  | 9, -88, -9 | R | | Lingual gyrus |
| 224 | 6.00 |  |  |  | -36, -94, -4 | L | | Cuneus |
| 1341 | 5.71 |  |  |  | -15, -84, -46 | L | | Cerebellum Crus II |
| 302 | 5.44 |  |  |  | -34, -30, -10 | L | | Hippocampus |
| 445 | 5.32 |  |  |  | -30, -62, -10 | L | | Fusiform gyrus |
| ***Semantic VF*** | | | | | | | | |
| 1268 | 7.77 |  |  |  | 20, -28, 4 | R | | Thalamus |
| 525 | 7.76 |  |  |  | 9, -3, 40 | R | | Cingulate gyrus |
| 1324 | 7.44 |  |  |  | -15, -86, -46 | L | | Cerebellum Crus II |
| 373 | 7.37 |  |  |  | -34, -30, -9 | L | | Hippocampus |
| 1588 | 7.23 |  |  |  | -32, 8, -2 | L | | Putamen |
| 436 | 5.58 |  |  |  | -38, -93, -4 | L | | Cuneus |
| 720 | 5.24 |  |  |  | 8, -88, -9 | R | | Lingual gyrus |

| Table Supplemental Table 4. Overview of the atrophy clusters (k≥50) and the affected structures. | | | | | | | | |
| --- | --- | --- | --- | --- | --- | --- | --- | --- |
| ***k*** | **Pseudo-t** |  |  |  | **MNI (x,y,z)** | **Hemisphere** | **Structure** | **Function** |
| 1409 | 6.97 |  |  |  | -4, -90, -12 | L | Calcarine Cortex | Primary visual cortex (V1) |
| 370 | 6.42 |  |  |  | -40, -64, 6 | L | Pericalcarine cortex | Associated visual cortices (V3-5) |
| 294 | 6.38 |  |  |  | -42, -90, -4 | L | Pericalcarine cortex | Associated visual cortices (V2-3) |
| 1507 | 5.68 |  |  |  | -63, -56, 2 | L | Medial temporal gyrus | Semantic & memory processes |
| 295 | 5.29 |  |  |  | 16, -54, -63 | R | Cerebellar tonsil (H IX) | Motor coordination & planning |
| 707 | 5.28 |  |  |  | -15, -86, -38 | L | Cerebellar Crus II | Rule processing |
| 250 | 5.25 |  |  |  | -63, -2, -16 | L | Medial temporal gyrus | Semantic & memory processes |
| 113 | 5.01 |  |  |  | -8, 14, 8 | L | Caudate nucleus | Goal-oriented behaviour |
| 59 | 4.82 |  |  |  | 57, 21, 4 | R | Pars triangularis | Semantic processing |
| 394 | 4.71 |  |  |  | -34, -70, -15 | L | Fusiform gyrus | Object & face recognition |

Table 4 shows the areas which atrophied over five years, and their associated functions.

**
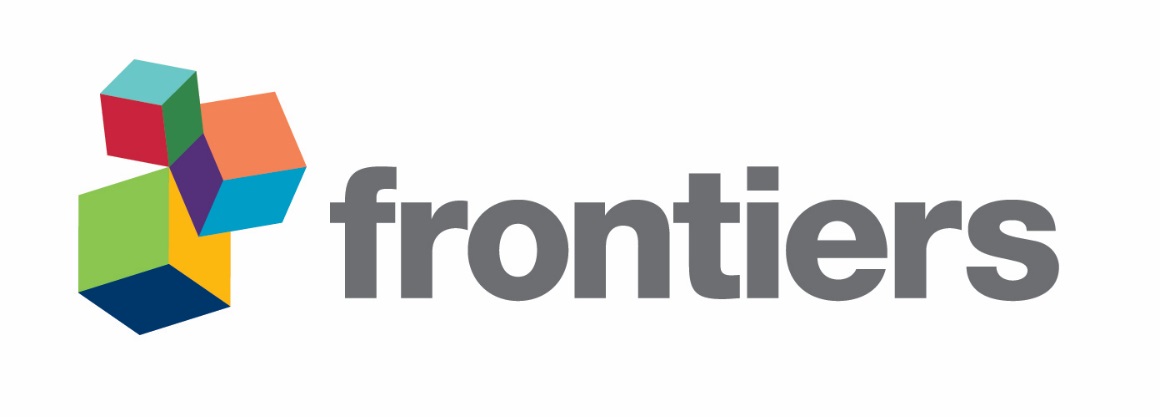
**

1. Abrahams S, Leigh PN, Harvey A, Vythelingum GN, Grisé D, Goldstein LH. Verbal fluency and executive dysfunction in amyotrophic lateral sclerosis (ALS). Neuropsychologia. 2000;38(6):734-47.
